# A xenograft animal model of human arteriovenous malformations

**DOI:** 10.1186/1750-1172-8-199

**Published:** 2013-12-30

**Authors:** Fang Hou, Yuemeng Dai, James Y Suen, Chunyang Fan, Ali G Saad, Gresham T Richter

**Affiliations:** 1 Center for the Investigation of Congenital Aberrancies of Vascular Development, Little Rock, AR, USA; 2 Department of Otolaryngology, University of Arkansas for Medical Sciences, Little Rock, AR, USA; 3 Department of Pediatric Surgery, Sichuan Academy of Medical Sciences/Sichuan Provincial People’s Hospital, Chengdu, Sichuan, China; 4 Department of Pathology, University of Arkansas for Medical Sciences, Little Rock, AR, USA; 5 Division of Pediatric Otolaryngology, Arkansas Children’s Hospital, 1 Children’s Way, Little Rock 72202, AR, USA

**Keywords:** Arteriovenous malformations, Animal model, Nude mouse, In vivo

## Abstract

**Background:**

Arteriovenous malformations (AVMs) are a type of high-flow vascular malformations that most commonly occurs in the head and neck. They are present at birth but are usually clinically asymptomatic until later in life. The pathogenesis of AVMs remains unclear and therapeutic approaches to AVMs are unsatisfied. In order to provide a tool for studying the pathogenesis and therapies of this disease, we established and studied a xenograft animal model of human AVMs.

**Methods:**

Fresh human AVMs specimens harvested from 4 patients were sectioned (5x5x5 mm) and xenografted subcutaneously in 5 immunologically naïve nude mice (Athymic Nude-Foxn1^nu^). Each mouse had four pieces specimens in four quadrants along the back. The grafts were observed weekly for volume, color and texture. The grafts were harvested at every 30 days intervals for histologic examination. All grafts (n = 20) were sectioned and stained for hematoxylin and eosin (H&E). Comparative pathologic evaluation of the grafts and native AVMs were performed by two blinded pathologists. Immunohistochemical examination of human-specific nuclear antigen, vascular endothelial growth factor receptor-2 (VEGFR-2) and Ki-67 was performed.

**Results:**

Clinical characteristics and pathologic diagnosis of native human derived AVMs were confirmed. 85% (n = 17) of AVM xenografts survived although the sizes decreased after implantation. Histological examination demonstrated numerous small and medium-size vessels and revealed structural characteristics matching the native AVMs tissue.76.5% (n = 13) of the surviving xenografts were positive for Ki-67 and human-specific nuclear antigen suggesting survival of the human derived tissue, 52.9% (n = 9) were positive for VEGFR-2.

**Conclusions:**

This preliminary xenograft animal model suggests that AVMs can survive in the nude mouse. The presence of human-specific nuclear antigen, VEGFR-2, and Ki-67 demonstrates the stability of native tissue qualities within the xenografts.

## Background

Arteriovenous malformations (AVMs) are a type of high-flow vascular malformations [1,2]. They are usually congenital and clinically asymptomatic until later in life. AVMs are known to arise from a cluster of aberrant arteriovenous shunts. They are comprised of numerous hypertrophic tortuous arteries that drain into arterialized veins forming a vascular nidus, which thought to have growth potential [3-5]. Although, AVMs are thought to grow synchronously with a child’s development, as a result of hormonal changes, inappropriate therapy and trauma, dramatic enlargement can occur [6-10]. This rapid and progressive enlargement will cause dysfunction, disfigure and even become a life-threatening problem [6,7,11]. Various therapeutic options have been used for AVMs, including surgical excision, surgical ligation of feeding arteries, selective vessel embolization, sclerotherapy and laser therapy. But none of the current therapeutic modalities is ideal for AVMs [6,7].

Despite many years of research, the etiology and pathogenesis of AVMs is not yet clear and the current therapeutic modality of AVMs is not yet satisfactory. In this study, with the aim of developing a reproducible model of AVMs and understanding the pathogenesis and exploring novel therapies of AVMs, we herein present preliminary results of an animal model of human AVMs by using human AVM tissue xenografting.

## Methods

### Specimens

This study was approved by the Institutional Review Board (University of Arkansas for Medical Sciences) and Institutional Animal Care and Use Committee (University of Arkansas for Medical Sciences). Fresh surgical specimens of head and neck subcutaneous AVMs were obtained from 4 patients (43-year-old female, 41-year-old female, 30-year-old male and 21-year-old female) with the diagnosis confirmed via clinical, radiographic and histologic assessment. At the time of resection, surgical specimens were divided for xenograft implantation and formalin fixation (10%) with paraffin embedding.

### The establishment of animal model

Five female 6-to 8-week-old nude mice (Athymic Nude-Foxn1^nu^; Harlan, Houston, TX) were used for this experiment. Ketamine hydrochloride (100 mg/mL; ButlerSchen, Dublin, OH), xylazine hydrochloride (40 mg/mL; AnaSed, Shenandoah, IA) and phosphate-buffered saline solution (PBS, pH 7.4; Sigma-Aldrich, St Louis, MO) at premixed 1:1:2.3 volume ratios were used for intraperitioneal injection anesthesia (2.22 mL/Kg) in mice. Fresh human AVM specimens were sectioned into approximately 5×5×5 mm pieces and inserted subcutaneously into 4 quadrants along the back of the nude mice. A single section was placed at 1 quadrant. Two mice were used for placement of the sections from the 43 year-old-patient. A total of 5 mice were used to place 20 xenografts in this experiment.

### Observation, measurement and harvesting

The xenografts were monitored for volume, color and texture at 7-day intervals throughout the period of the study. Simultaneously, the size of each graft was recorded using vemier calipers and using the average of the 2 largest diameters (a and b). The volume (V) of grafts was estimated as V = π/6[(a × b)^3/2^] [12]. With satisfactory anesthesia, each graft was harvested at essentially serial 30-day intervals (30, 60, 90, and 120 days). All mice were humanely killed after removing the final graft.

### Histological analysis

All the harvested grafts were fixed in 10% neutral buffered formalin and embedded in paraffin. Then four-micrometer thickness sections were cut, deparaffinized and stained with hematoxylin (Fisherbrand, Pittsburgh, PA) and eosin (Thermo Fisher Scientific, Waltham, MA) (H&E). All the slides were examined by 2 blinded pathologists (CY. Fan and A.G. Sadd) experienced with vascular anomalies (VA).

### Immunohistochemistry

After deparaffinization and rehydration, the sections were heated to 97°C for 20 minutes in a water bath in the presence of antigen retrieval solution (CITRA, pH 6.0; Invitrogen, Carlsbad, CA) and cooled for 30 minutes. To block the endogenous peroxidase activity, all sections were incubated with hydrogen peroxide for 10 minutes and washed with a PBS (pH 7.4; Sigma-Aldrich, St Louis, MO). Sections were preincubated with 2% nonfat milk for 30 minutes at room temperature. Then sections were incubated with primary Vascular endothelial growth factor receptor-2 (VEGFR-2, rabbit anti-human polyclonal antibody; Abcam, Cambridge, MA) at 1:200 dilution or Human-specific nuclear antigen (mouse anti-human monoclonal antibody; Millipore, Billerica, MA) at 1:400 dilution or Ki-67 (rabbit anti-human monoclonal antibody; Abcam, Cambridge, MA) at 1:100 dilution for 20 hours at 4°C. After washing in PBS, sections were incubated in primary antibody enhancer (Thermo Fisher Scientific, Waltham, MA) for 10 minutes and horseradish peroxidase polymer (Thermo Fisher Scientific, Waltham, MA) for 15 minutes at room temperature. After washing the sections in PBS, they were incubated with diaminobenzidine (Thermo Fisher Scientific, Waltham, MA) for 5minutes at room temperature. The sections were counterstained with hematoxylin for 20 seconds. Sections were dehydrated through graded alcohol solutions and cleaned by xylene substitute. Then they were mounted with permount (Thermo Fisher Scientific, Waltham, MA) and coverslipped.

Native human AVM specimens were used as a positive control for VEGFR-2 antibody. Human tonsil tissues were used as a positive control for Ki-67 antibody. Unaffected mouse skin and subcutaneous tissues were used as the negative control for VEGFR-2 antibody. Mouse myocardium tissues were used as the negative control for Human-specific nuclear antigen. The staining results were validated by a blind review performed by 2 pathologists (CY. Fan and A.G. Sadd) with extensive experience examining VA and immunohistochemistry. A strong staining in greater than 10% of the cells indicated a positive value.

## Results

### Xenograft quality

**Figure 1:**
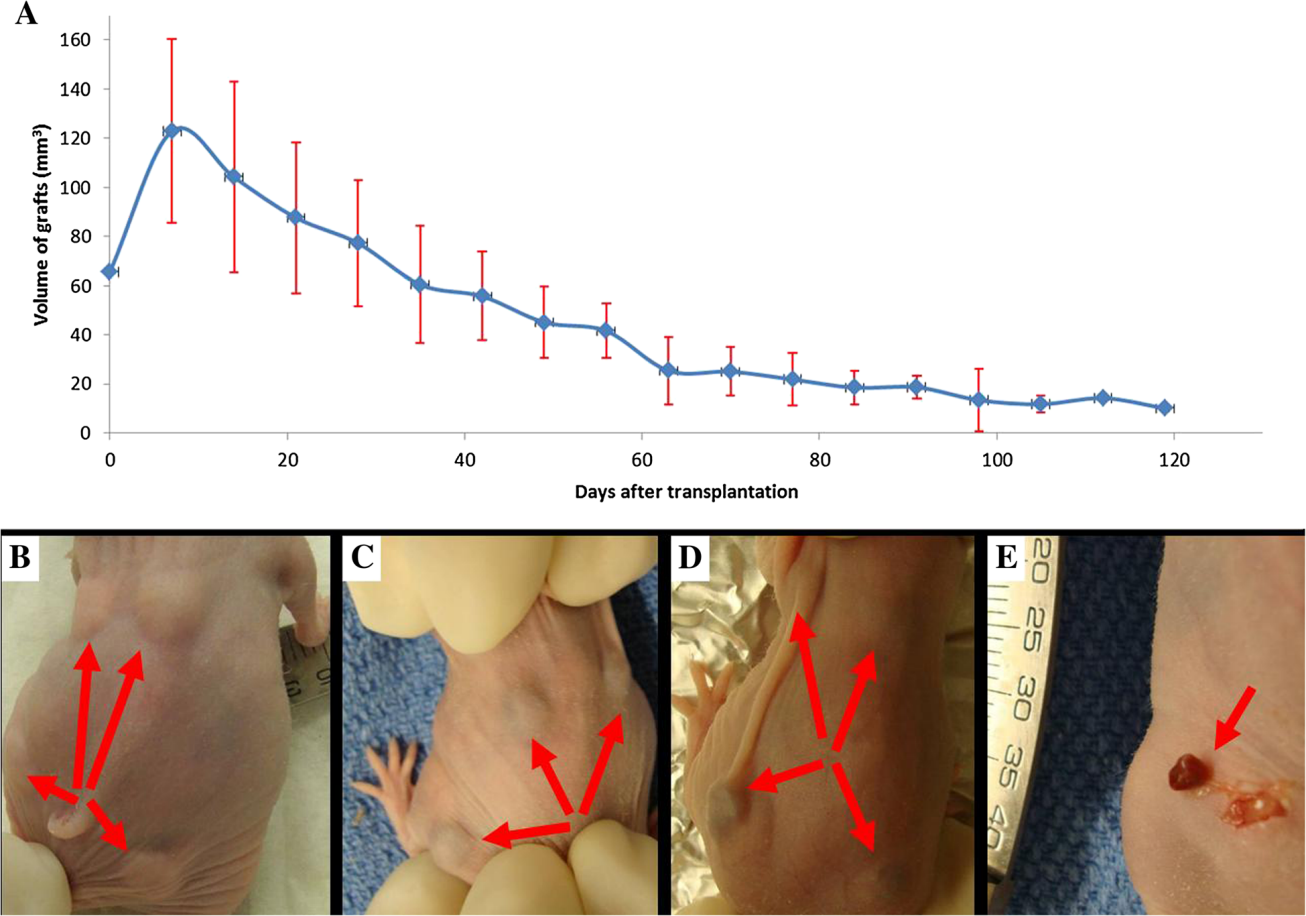
**The growth characteristics of human AVM xenografts in nude mice. (A)** The general drift of the grafts voluminal alteration after transplantation (mean ± SD). **(B)** Graft appearance in the four quadrants of the nude mouse back at 5 days post transplantation (arrows). **(C)** 18 days after transplantation, the grafts turn to blue discoloration and a harder texture (arrows). **(D)** At 27 days, the grafts gradually turned purple (arrows). **(E)** At 60th day harvest, the graft was vascular and slightly smaller in size than original tissue (arrow).

All nude mice survived the entire experimental protocol until the last graft was harvested. On average, the xenografts had a small but brief growth phase until day 30–40. Following this transition the xenografts underwent a gradual decrease in size. Graft size appeared quiescent from the 60^th^ to 95^th^ day after transplantation (Figure 1A, The data were expressed as means ± SD). Before transplant, the fresh human AVM specimens were red and consistently soft in texture. After transplantation, the grafts appearance under the nude mouse skin was pink, flat and soft until day 18 of transplantation with a shift to blue discoloration and a harder texture (Figure 1B, C Arrow). At 1 month, the grafts gradually turned purple (Figure 1D, Arrow). At the date of harvest, 18 harvested grafts (90%) were round and vascular (Figure 1E, Arrow).

### Histopathology of grafted AVMs

**Figure 2:**
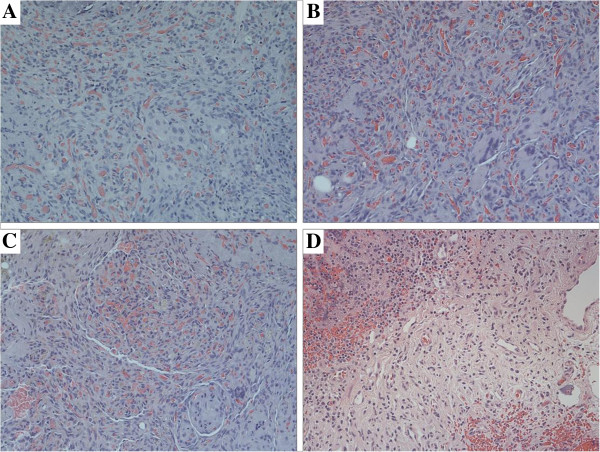
**Histopathology of xenograft AVMs.** (HE × 200). 30 days **(A)**, 60 days **(B)**, 90 days **(C)** after transplantation, abundant of plump ECs with inconspicuous lumina were crowded together, in the center of the tissue. The densely of cells and vessels were almost maintained despite day of harvest. ECs in the grafts which are characterized by large nuclei and scant cytoplasm, lining inconspicuous vessels can be found has the same structural characteristics as the ECs in the pre-implanted human AVM nidus **(D)**.

H&E staining of the harvested xenografts sections showed 17 grafts (85%) were composed of densely packed vessels and presence of a vascular malformation despite day of harvest (Figure 2A, B, C). This suggest those grafts were survived throughout the experiment.

**Figure 3:**
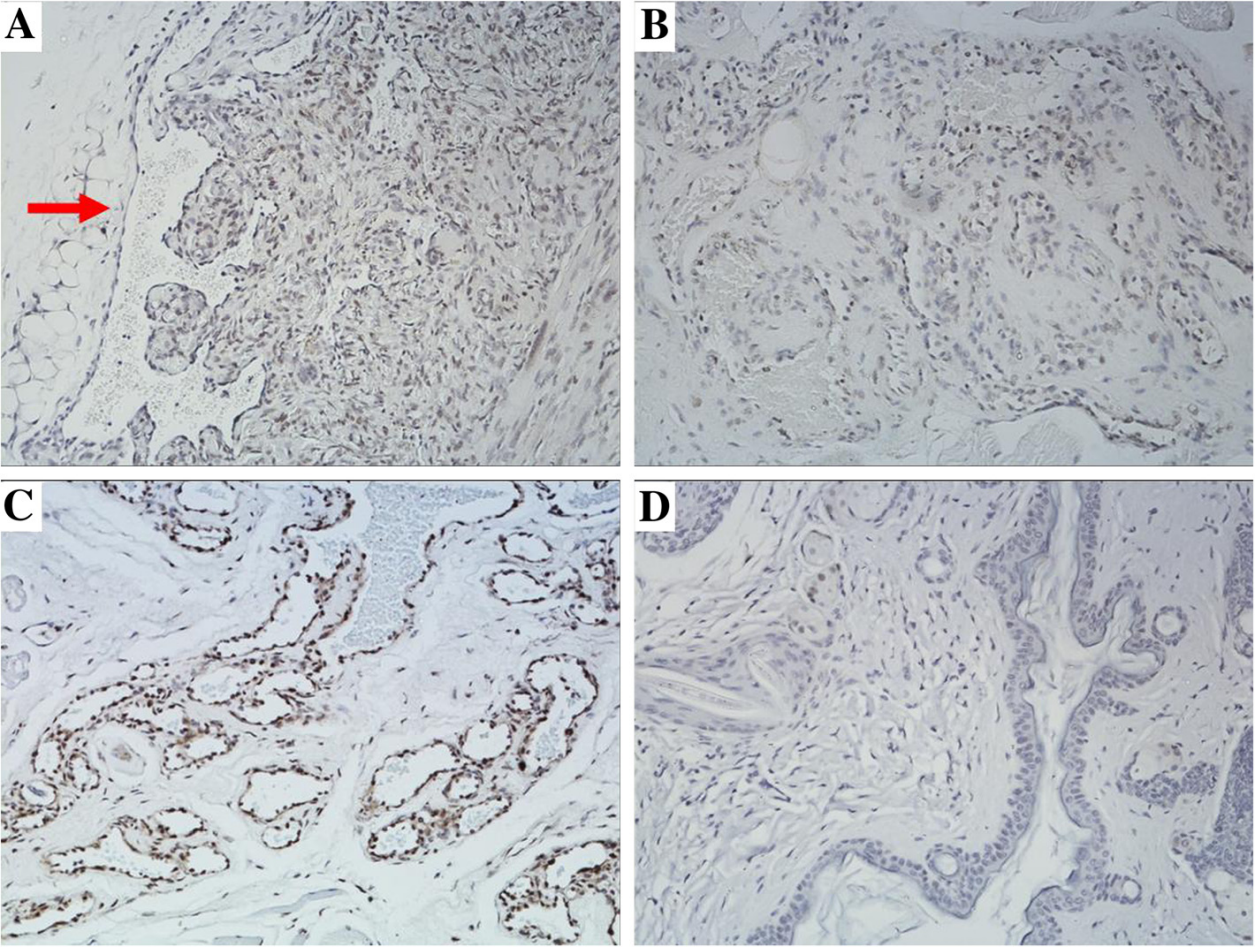
**The immunostaining for VEGFR-2.** (IHC × 200). **(A)** A lot of AVM “nidus” ECs in the grafts were positive for VEGFR-2. Tortuous thick-walled arteries and veins were noted in the peripheral area (arrow). **(B)** The ECs in the pre-implanted human AVM “nidus” were positive for VEGFR-2. **(C)** The ECs of arteries and veins in pre-implanted human AVM specimens were positive for VEGFR-2. **(D)** Unaffected mouse skin and subcutaneous tissues were negative for VEGFR-2.

Especially, in the center of the xenograft sections, abundant of plump endothelial cells (ECs) with inconspicuous lumina were crowded together which has the same structural characteristics of human AVM nidus (Figure 2). While, in the peripheral area, the identification of tortuous thick-walled arteries and veins were noted (Figure 3A, Arrow).

Generally, tissue quality was better in younger grafts. Thirty days after transplantation, in the center of the tissue, the cellular density was highest. At 60 days after transplantation fibrous and fat tissue was identified in the harvested specimens, which was retained until day 120. Although the grafts decreased in volume with time but the densely of cells and vessels were almost maintained (Figure 2A, B, C).

### Immunohistochemistry

To verify that the AVMs in the xenograft were retained human specimens and composed of human ECs, we performed Immunohistochemistry by using Human-specific nuclear antigen and anti-human VEGFR-2 antibody.

**Figure 4:**
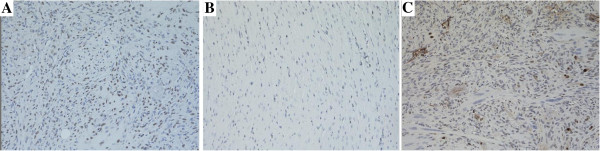
**AVM grafts immunostaining for Human-specific nuclear antigen and Ki-67.** (IHC × 200). **(A)** Human-specific nuclear antigen was positive in the grafts, but negative **(B)** in Mouse myocardium tissues. **(C)** Ki-67 was positive in transplanted grafts.

A total of 76.5% grafts (n = 13) were positive for Human-specific nuclear antigen (Figure 4A) and 52.9% (n = 9) grafts were positive for human VEGFR-2 (Figure 3A), of the survived xenografts (n = 17). The pre-implanted human specimens of AVMs were confirmed positive for VEGFR-2 immunostaining (Figure 3B, C). Unaffected mouse skin and subcutaneous tissue was also stained and confirmed negative for VEGFR-2 (Figure 3D), and mouse myocardium was negative for Human-specific nuclear antigen (Figure 4B).

To further examine the AVMs in the xenograft have the potential to grow, we immunostained for Ki-67, a marker of cellular proliferation. Of the survived xenografts, 76.5% grafts (n = 13) were positive for Ki-67 (Figure 4C). But, the pre-implanted human AVM tissues were confirmed negative for Ki-67 immunostaining.

## Discussion

AVMs tend to progress slowly, but prior therapy, pregnancy or trauma may cause their rapid expansion [6-10]. With time, AVMs will expand to excessive size and infiltrate local tissues. This relentless growth will cause mass effect on surrounding tissues, lead to functional deficits, aesthetic impairment, long-term sequelae and even become a life-threatening problem [6,7,11]. Unfortunately, current therapeutic modalities are unsatisfied; especially many treatments have shown to stimulate the AVMs recruitment [6,7].

Some evidence suggests that complete surgical resection with preoperative embolization has remained the most likely chance for cure, especially in the early phase [7,13]. But diagnosis of such lesions in the early phase is not easy. Many AVMs are misdiagnosed as infant hemangiomas (IH) during infancy which will limit patient access to appropriate therapy [7]. The problematic aspect of these lesions has been compounded by the etiology and pathogenesis of AVMs is poorly understood. This has led to invalid and wrong treatment protocols [14-17]. Besides, the research of AVMs at present mostly depends on the study of surgical specimens. To understand development of AVMs and improve the treatment, animal models for the disease have to be developed.

To date, there are no animal models that truly reflect the human AVMs. Experimental models of brain AVMs have been established in rats, cats and dogs [18-21]. Especially, rat models have been most extensively investigated. It was reported that the model in rat has been created by anastomosing the caudal end of the external jugular vein to the side of the common carotid artery [21]. But all these models represent Arteriovenous fistulae rather than AVMs. Besides, these models were a surgically created and do not contain the native human AVM nidus [21].

Recently, Yao et al., [22] reported a very interesting AVM model by using Mgpnull (Mgp-/-) mice. But Matrix Gla protein (Mgp) gene deletion in mice only formatted AVMs in lungs and kidneys. And mice that lack of Mgp will die within two months as a result of arterial calcification which leads to blood-vessel rupture [23]. While, human AVMs are usually thought to be a kind of chronic and slow development disease. More importantly, these models were not developed from native human AVMs, the ideal tissue sample for investigation of this disease.

Previous studies have demonstrated that the IH and lymphatic malformations, two different types of VA, can survive in the nude mouse [24-26]. Nude mice are thymus absent and T-cell deficient. They have the ability to receive many different types of tissue and tumor xenografts [27-31]. As another type of VA, we hypothesized that AVMs may also survive in the nude mouse and likely to retain their principal morphologic and biological properties when grafted. To test this hypothesis, we used nude mouse as the vehicle to establish a human AVM tissue xenografted model.

In this study we demonstrate the survival of human AVMs in 17 of 20 (85%) xenografts harvested from 4 patients. A brief period of growth occurred early but was not maintained as the samples became quiescent and subsequently diminished in size over a period of 120 days. Xenografted tissue appears ideally reflective of native AVMs at 20–30 days post transplantation. Necrosis or tissue loss was not identified in any of the harvested grafts. Structural characteristics of human AVMs were retained and confirmed by pathologic examination. In the survived grafts, abundant of plump ECs with inconspicuous lumina were crowded together just has the same structure as human AVM nidus which is characterized by large nuclei and scant cytoplasm, lining inconspicuous vessels. And also the tortuous thick-walled arteries and veins were found packed at the periphery of the specimens. These results suggest that xenografted human AVM tissues can survive in the athymic nude mice. However, AVMs cannot be identified only by light microscopic definition in current pathologic practice. It also needs to be confirmed by immunostaining. But at present, no specific markers that can labels AVM ECs and reliably diagnose AVMs.

VEGFR-2, as the EC marker, plays a broader role in angiogenesis. Its activity regulates endothelial cell growth, differentiation, migration and tubulogenesis; [32] While it has been used to identify the ECs in many studies, especially in AVMs and IH [33,34]. So, in this experiment, we choose anti-human VEGFR-2 antibody as a marker of AVM ECs. We have shown that, both the AVM ECs in the grafts and in the pre-implanted human AVM tiusses revealed strong positive staining of VEGFR-2. While the mice skin and subcutaneous tissue were confirmed negative for VEGFR-2 immunostaining. To verify that the AVMs in the mice xenografts were composed of human ECs, we performed staining specific to human antigens. The strong positive staining of Human-specific nuclear antibody grafts are the majority of our samples (76.5%). These findings indicate that the AVMs in the mice xenografts originated from human ECs and were not the result of infiltration by murine ECs.

AVMs are thought to have nidus, packed with immature capillary vessels, which is the angiogenesis center of AVMs. It has inherent growth potential and high level of angiogenic factors [3-5]. In this research, only 52.9% (n = 9) of implants in all the survived xenografts were positive for VEGFR-2. While 76.5% (n = 13) of mice xenografts have been shown originated from human. We speculate that some specimens we have plant into nude mouse are thin-walled small mature AVM vessels instead of the AVM nidus.

In this research, to identify the potential presence of proliferating cells within the xenografts, we immunostained for Ki-67, a marker strictly associated with cell proliferation [35]. Although AVMs are a slow growing entity in humans, Ki-67 positive AVM ECs have been documented in many studies [4,36,37]. Interestingly, we found that 13 grafts stained positive for Ki-67, while the original human AVM specimens were negative for Ki-67. Also we have observed that the xenografts had a brief growth phase until day 30–40 after transplantation. These results may indicate that cellular proliferation is occurring in the tissue sample following transplantation, which is consistent with the fact that AVMs often clinically silent, but surgery, trauma and hormonal changes may trigger their rapid expansion [6-10].

In addition, this study had a number of limitations. First, although the xenografts had a small but brief growth phase, they were found to slowly but progressively decrease in size after day 30–40 post transplantation. One possible explanation for this is the transplantation may only trigger the AVMs expansion for a short phase without human hormones. Second, we can not tell the original human AVM specimens, we have plant into nude mouse, are mature AVM vessels or the AVM nidus. Maybe the growth of the xenografts will be sustained when they contain dominant AVM nidus. Third, AVMs as a kind of chronic and progressive disease, it usually tends to progress slowly. However, the protocol of this study required that the grafts be harvested at 30 day intervals until harvested them all (120 day max). How long these AVM grafts can survive, and if they can be stimulated to grow, needs further research.

Although this model may does not accurately reflect all of the features of human AVMs, this study reveals for the first time that human AVMs can survive in nude mouse with structural and molecular characteristics similar to the original human specimens. This in vivo model is unique in that the AVM lesions are able to observe by the naked eye, and it may provide a valuable resource for examining the pathophysiology and potential treatment.

## Abbreviations

(AVMs): Arteriovenous malformations; (H&E): Hematoxylin and eosin; (VEGFR-2): Vascular endothelial growth factor receptor-2; (PBS): Phosphate-buffered saline; (VA): Vascular anomalies; (ECs): Endothelial cells; (IH): Infant hemangiomas; (Mgp): Matrix Gla protein.

## Competing interests

The authors declare that they do not have any competing or financial interests.

## Authors’ contributions

FH and GTR conceived and designed the experiments. FH and YD performed the experiments. FH, CF, GTR and AGS analyzed the data. FH, GTR and YD contributed reagents/materials/analysis tools. FH and YD wrote the paper. GTR and JYS critical revision of the manuscript for important intellectual content. All authors read and approved the final manuscript.

## Previous presentation

This study was presented at the 19^th^ International Society for the Study of Vascular Anomalies, 2012 June, Malmo, Sweden.
